# P-1748. Reducing Inappropriate Post-Operative Antibiotics in Pediatrics: The Optimizing PERioperative AnTibiotics In Children (OPERATIC) Trial

**DOI:** 10.1093/ofid/ofae631.1911

**Published:** 2025-01-29

**Authors:** Jason G Newland, Jingxia Liu, Jade Tao, Jacqueline M Saito, Shawn Rangel, Kelly Bono, Sara Malone, Virginia McKay

**Affiliations:** Washington University in St. Louis School of Medicine, St. Louis, Missouri; WUSM, Saint Louis, Missouri; Washington University in St. Louis, St. Louis, Missouri; Children's National Hospital, Washington, District of Columbia; Boston Children's Hospital/Harvard Medical School, Boston, Massachusetts; Washington University School of Medicine, Saint Louis, Missouri; Washington University School of Medicine, Saint Louis, Missouri; Washington University in St. Louis, St. Louis, Missouri

## Abstract

**Background:**

Prolonged ( > 24 hours) post operative antibiotic (abx) use is the most common reason for inappropriate abx use in hospitalized children occurring in 40% of cases. Effective strategies are needed to reduce inappropriate post operative abx use. One strategy, facilitation, helps people develop the skills to change the structure and processes within a system to reduce the gap between evidence and practice. This study evaluated the best strategy, order set change vs order set change + facilitation, to deimplement unnecessary post operative abxs.

**Figure 1:**
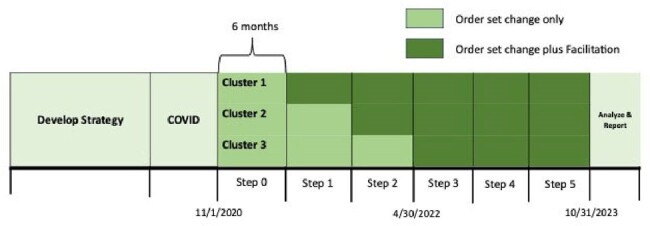
Operatic Stepped-Wedge Trial Design

Stepped-wedge trial design comparing order set change only (control) versus order set change plus a facilitation workshop (intervention) attended by the ASP team at the beginning of step. Nine children’s hospitals were randomized to 1 of 3 clusters to begin the intervention at either steps 1, 2, or 3. All hospitals were in the intervention in steps 3-5. Each cluster included 3 hospitals and each step was 6 months in duration.

**Methods:**

A stepped-wedge trial was conducted at 9 US children’s hospitals from 11/1/20-10/31/23 (Fig 1). After 6 months of all hospitals starting in control of changing order sets, 3 hospital antimicrobial stewardship teams in steps 1-3 participated in the intervention (int), a virtual facilitation workshop to help change order sets. The primary outcome was the rate of inappropriate post operative abxs ( > 24 hours) in clean and clean contaminated cases. Hospital data were collected through the National Surgical Quality Improvement Project Pediatrics. Secondary outcomes were rate of surgical site infection (SSI) and *C. difficile* infection (CDI). Generalized estimating equations (GEE) with logit link function were utilized to investigate the outcome changes across steps.

**Figure 2.**
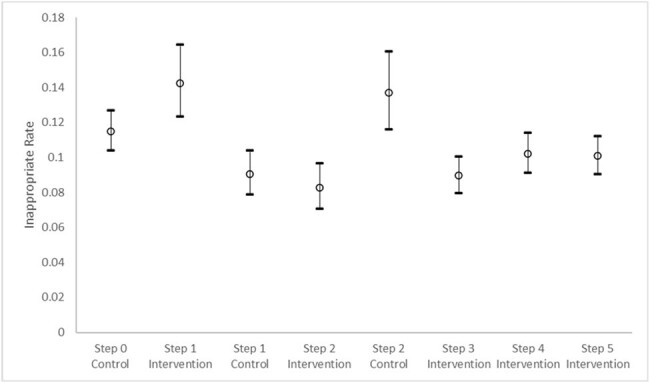
All Cases

All NSQIP-P clean and clean-contaminated cases inappropriate rate of post-operative antibiotic use (>24 hours) by step and group (control and intervention). The GEE model of inappropriate antibiotic rate is presented as the least square means with 95% Confidence intervals.

**Results:**

The study included 17202 cases, 5913 control and 11289 int. Demographics included mean age of 8.4 years (sd 5.9); 51% female; 71% White, 14% Unknown, 9% Black race; and 14% Hispanic ethnicity. The common surgical specialties were orthopedics (31%), general (21%), and neurosurgery (17%). The overall post operative inappropriate rate was 10%, 11% control v. 10% int. 4 hospitals targeted spine cases (n 2733); the inappropriate rate was 28%, 33% control v. 26% int. Controlling for time and hospital, the GEE model showed a significant decrease in inappropriate post operative abx use for all cases and spine cases for the int across steps (Fig 2&3 p< 0.001). SSI and CDI rates were not significantly increased or decreased.

**Figure 3.**
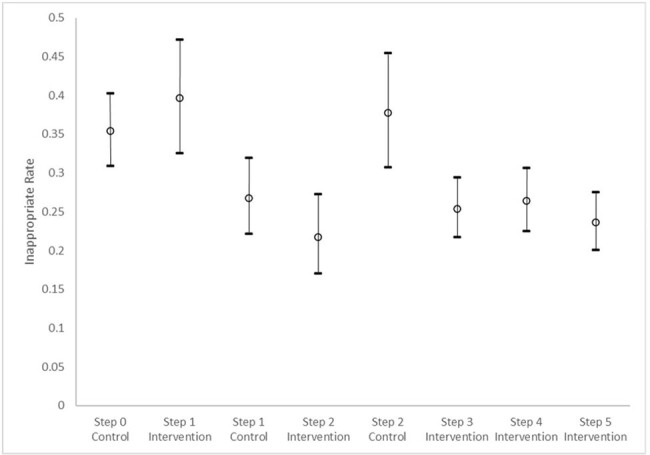
Spine Cases Spine cases inappropriate rate of post-operative antibiotic use (>24 hours) by step and group (control and intervention). The GEE model of inappropriate antibiotic rate is presented as the least square means with 95% Confidence intervals.

**Conclusion:**

Order set change plus facilitation resulted in a significant reduction in inappropriate post operative abx use. This study is the first deimplementation trial to show the impact of a facilitation workshop on inappropriate abx use.

**Disclosures:**

**Jason G. Newland, MD, MEd**, Moderna: Grant/Research Support|Pfizer: Grant/Research Support

